# Effect of Boiling on the Nutrient Composition of *Solanum Torvum*

**DOI:** 10.1155/2022/7539151

**Published:** 2022-03-16

**Authors:** Janice Dwomoh Abraham, Emmanuel Kwadwo Sekyere, Isaac Gyamerah

**Affiliations:** ^1^Department of Biological Sciences Education, Faculty of Science Education, Akenten Appiah-Menka University of Skills Training and Entrepreneurial Development, Mampong-Ashanti, Ghana; ^2^Department of Integrated Science Education, Faculty of Science Education, Akenten Appiah-Menka University of Skills Training and Entrepreneurial Development, Mampong-Ashanti, Ghana

## Abstract

The fruits and leaves of *Solanum torvum* are good sources of nutrients and minerals for the prevention of nutrient deficiencies. However, there is limited information on the effect of boiling on the nutrients, minerals and phytochemicals in the fruits and leaves. This study sought to assess the mineral, macronutrient and phytochemical compositions of fresh and boiled fruits and leaves of *S. torvum*. Fresh unripe fruit and leaf samples of *S. torvum* were collected from six communities, boiled, and pulverized for mineral, proximate and phytochemical analyses. The data obtained was subjected to ANOVA and t-test. *Solanum torvum* was found to contain Fe, Zn, Cu, Mn, Ca, Mg, Na, K, protein, crude fat, carbohydrate, fibre, saponins, tannins, flavanols, terpenoids/steroids and glycosides making it nutritious. The results revealed almost equal concentrations of minerals in fresh and boiled leaves and fruits. A similar observation was made in the case of carbohydrate, crude fat and ash. However, there were significant differences in moisture, protein and crude fibre concentrations in the samples. Phytochemical analysis revealed the presence of saponin, tannin, flavonoids, terpenoids/steroids and glycosides in all leaf samples no matter the treatment. There were no flavonoids and terpenoids/steroids in fruits. Boiling nominally reduced and in a few cases, increased concentration of the nutrient composition but did not have significant effect on the concentration of the macro- and micro- minerals in the fruits and leaves. This study suggests that boiling could affects the concentration of nutrients that could be accessed in fruits and leaves of *S. torvum*.

## 1. Introduction

Nutrition is very crucial to life because it enhances metabolism and build the immune system of organisms including humans. Research shows that micro-minerals such as Fe, Zn, Cu and Mn are essential for the physiological function of organisms and thus deficiency of these may damage DNA, cause stunted growth, affect enzyme action, blood Hb concentration and functioning of organs such as the eye and brain [1–3]. Unfortunately, the effect of malfunctioning organs is only realized during the later ages of affected persons [2–4]. Phytochemicals such as saponins and glycosides are anti-cancerious and antibiotic compounds that help to prevent conditions such as metabolic malfunction and growth deficit [5, 6]. These chemicals are obtained mostly from plants sources. The daily diet of tropical African countries is often dominated by starchy staples and coarse grains which provide energy but do not support growth, metabolic functioning and immune defense of the individual [2, 7]. Indigenous vegetables such as *Amaranthus cruentus*, *Celusia argenta* and *Corchorus olitorius* are the cheapest and most readily available sources of proteins, vitamins, minerals and essential amino acids for many people [8, 9]. Vegetables such as *Digera arvensis, Solanum nigrum* have high nutritional value [9–12]. Thus, their adequacy in a diet would help increase the supply of some nutrients that might be absent in people's regular diet [13, 14]. In Africa, wild edible plants are used as food and hence contribute significantly to the nutritional needs of the people [9, 15]. *Solanum torvum* commonly called turkey berry is one of those wild plants known to be helpful in nutrition. The plant is utilized as vegetable and regarded as an essential ingredient in the diet of South Indians [16]. Its leaves are eaten as a leafy vegetable and the fruits eaten raw or cooked. Phenolic compounds extracted from different parts of *S. torvum* exhibited anti-oxidant activity [9, 17]. It is also reported that the plant has anti-mycobacterial and cytotoxic activities [18]. In Ghana, *S. torvum* is locally referred to as “Kwahunsusua*”*, “Yaa Asantewaa” or “Abeduru” and is used for the preparation of vegetable stew and soup. One of the soups prepared with *S. torvum* is “abeduru” and is usually given to nursing mothers in southern Ghana to aid lactation and enhance recovery of mothers within few weeks of childbirth (JD Abraham per. obs.). It is generally believed in the Ghanaian society that the fruits of *S. torvum* are rich in minerals which help to improve blood conditions in humans [19, 20], hence, the use of its juice by local people for the management of anaemic condition especially, in pregnant women and children [21]. The juice from the fruit is used as blood tonic probably because of its Fe content in some traditional homes [21]. To the best of our knowledge, very little scientific evidence is documented in the Ghanaian literature to support the claim that *S. torvum* improves blood levels [22]. Additionally, there is very little information on the effect of boiling on nutrient composition of *S. torvum*. Therefore, this study investigated the effect of boiling on the nutrient composition of the fruits and leaves of *S. torvum.*

## 2. Materials and Methods

### 2.1. Plant Material

**Figure 1:**
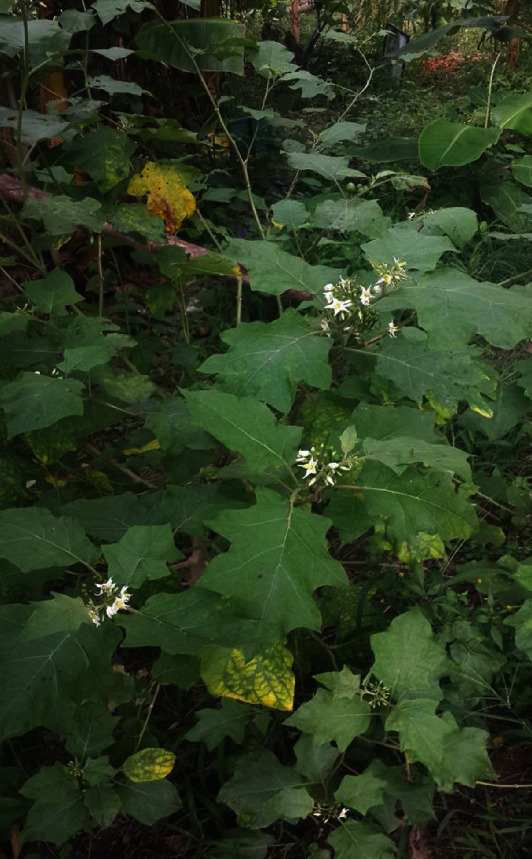
A matured flowering *Solanum torvum* plant. Photo credit: Emmanuel Kwadwo Sekyere.

*Solanum torvum* (Figure 1) is an erect spiny shrub about 2-4 m tall with many branches and large thorny impenetrable thicket [23]. The stem and branches are sparsely prickly. The leaves are petiolate, simple, solitary and alternate or in unequal pairs or triplets, ovate to lanceolate and about 7.5-25 cm long. The leave margins are entirely to shallowly lobed [24]. The plant has small star-shaped white flowers with broadly triangular to ovate-lanceolate sepal lobes which occur in large clusters [25]. The fruits are small (about 1 cm in diameter), succulent, green and globose in shape. The fruits contain numerous flat, round, brown seeds and turns yellow when fully ripe [25, 26].

### 2.2. Sample Collection and Preparation

Fresh unripe fruits and leaves of *S. torvum* were collected from the wild of six communities (i.e., Bonkuru, Kwamang, Nsuta, Krobo, Mampong and Ninting) in the Mampong Municipality and Sekyere Central District in the Ashanti Region of Ghana. Mampong Municipality is located within latitude 7° 4' 0“ N and longitude 1° 24' 0” W. Sekyere Central District is also located within latitude 7° 1' 0“ N and longitude 1° 22' 60” W.

The fruits and leaves were washed and divided into two equal parts. One half of the sample was boiled for 20 minutes while the other was not boiled. The fresh and boiled samples were labeled and stored at -21°C (Chest freezer, Zhengzhou Hepo International Trading Co., Ltd., China) for 3 – 5 days awaiting freeze drying, nutritional and phytochemical analyses.

For nutrient analysis, the samples were freeze-dried using a Drawell Freeze-drier (Shanghai Drawell Scientific Instrument Co., Ltd. Shanghai, China) at a cold trap of -46.7°C and left for 48 hours. The dried samples were pulverized using a blender (Kenwood Ltd, Tokyo, Japan) and stored in an airtight zip lock bag, wrapped with aluminum foil, labeled and stored at -21°C in the Chest freezer for 3 – 5 days awaiting analyses. The nutrient analysis was done at the Chemistry laboratory at the Soil Research Institute of the Centre for Scientific and Industrial Research, Kwadaso - Kumasi, Ghana and the phytochemical analysis was carried out in the Science laboratory of the College of Agriculture Education of the Akenten Appiah-Menka University of Skills Training and Entrepreneurial Development - Ghana.

### 2.3. Sample Analysis

The mineral analysis was done by weighing (Mettler Toledo, Shanghai, China) 0.5 g of the sample into a polystyrene microwave digester. An amount of 5 ml of concentrated nitric acid (HNO_3_) was added to the weighed sample in the digester bottle. The samples were then digested with a microwave digester system (Topex KJ-100, PreeKem, Shanghai, China) at a temperature of 180°C for 10 minutes. The digested sample was allowed to cool at room temperature and the content filtered (Ø 0.125 mm) through Whatman's filter paper (GE Healthcare Bio-Sciences, Pittsburgh, PA) into a 100 ml volumetric flask. The filtrate was topped with distilled water to the 100 ml mark. The aliquot was used to assess the concentration of Fe, Cu, Zn, Mn, Ca, Mg, P, S, Na and K using Atomic Absorption Spectrophotometer (AAS) (Agilent, 200 series AA System, California, USA) and Spectrophotometer (Shimazu: UV – 1800 series, Japan).

### 2.4. Determination of cu, Fe, Zn, Mn, ca, mg, Na and K

About 25 ml of the *S. torvum* aliquot was measured into volumetric flask. The concentration of Cu, Fe, Zn, Mn, Ca, and Mg in the aliquot was analyzed using the AAS at wavelengths of 324.7 nm, 248.3 nm, 213.9 nm, 279.5 nm, 422.7 nm and 285.2 nm, respectfully.

### 2.5. Determination of Macro-Minerals: Potassium (K) and Sodium (Na)

Using the aliquot of *S. torvum*, 1.0 ml of the sample was taken and 2.5 ml of lanthanum cesium oxide added to it. This was topped with distilled water to 25 ml. The concentration of ‘K' and ‘Na' were read using flame of the Atomic Absorption Spectrophotometer (AAS) at wavelength of 589 nm and 766.5 nm respectfully.

### 2.6. Determination of Phosphorous (P)

Using the colorimetric method, 0.5 g of the pulverized sample was placed into a polystyrene microwave digester bottle and 5 ml of concentrated nitric acid (HNO_3_) added to it for digestion in a microwave digester (Topex, Preekem, Shanghai, China) at a temperature of 180°C for 10 minutes. It was left to cool and the content filtered into 100 ml volumetric flask. After the filtration, 10 ml of colour reagent (ammonium molybdate, ammonium metavanadate and sulphuric acid) was added to the filtrate. The aliquot was topped with distilled water to the 100 ml mark from which 50 ml was taken for the assessment of phosphorus in the sample using spectrophotometer. The absorbent of the extracted sample was read with the aid of spectrophotometer at a wavelength of 420 nm.

### 2.7. Determination Sulphate (SO_4_)

The turbidimetric method was used to access the sulphate content in the samples. An amount of 5 ml of the *S. torvum* aliquot was measured into a volumetric flask and 10 ml of sodium acetate buffer solution added to it. After this, 1.0 g of barium chloride (BaCl_2_) was added and topped to the 25 ml with distilled water. The relative absorbance was read using spectrophotometer at wavelength 440 nm.

## 3. Proximate Analysis

### 3.1. Moisture Content

Moisture content in the sample was determined by a modified method of the Association of official Analytical Chemists (AOAC). Two grams of the samples were weighed and dried in a freeze drier until a constant weight was obtained. The final weight of the sample was taken from the initial weight to obtain the moisture content of the samples. After the drying, 2 g of the sample was placed into a clean, dry and weighed glass crucible. The crucible with its content was put into a drying oven (Wagtech Projects Ltd, Thatcham, UK) at 105°C for 6 hours until a constant weight was obtained. The loss in weight expressed as a percentage of the initial weight of sample gave the percent moisture content using the formula.

% Moisture = ×100%

(1) $$\begin{array}{c} \%\text{moisture}=\frac{\text{weight of wet sample}-\text{weight of dry sample}}{\text{Weight of wet sample}}100\% \end{array}$$

### 3.2. Ash Content

Ash was determined by the method of the association of the official analytical chemists (AOAC). Clean empty Porcelain crucibles were placed in a Muffle furnace (Witeg Labortechnik GmbH, Wertheim, Germany) preheated at 600°C for an hour, cooled in desiccators. The crucible was then weighed and the initial weight recorded. A gram of each sample was weighed into the previously dried and weighed porcelain crucible. The crucibles with it content were placed in a Muffle furnace preheated at 600°C for two hours. After this period, the crucibles with its content were removed and cooled in desiccators. The crucibles with their content were weighed. The weight of the ash was expressed as a percentage of the initial weight of the sample % Ash = × 100% using the formula.

(2) $$\begin{array}{c} \%\text{Ash}=\frac{Difference\;\text{in }Weight\;\text{of }Ash}{weight\;\text{of }sample\;}\times 100\% \end{array}$$

### 3.3. Crude Protein Content

The crude protein content of the sample was determined by the kjeldahl method. One gram of dried samples was placed into a digestion flask after which 15 ml of concentrated H_2_SO_4_ and 8 g of digestion mixture (K_2_SO_4_ CuSO_4_ (8 : 1)) were added. The flask was swirled to get a uniformed mixture. The flask was then placed on a digestion burner for 2 – 3 hours to digest the mixture to get clear solution (blue-green in colour). The digest was cooled and transferred into 100 ml volumetric flask. The digest was topped with distilled water to the 100 ml mark. After that, 25 ml of 2% boric acid was pipetted into a 250 ml conical flask and three (3) drops of mixed indicator (made up of 20 ml of bromocresol green and 4 ml of methyl red) solution was added to it. After that, 10 ml of the aliquot was measured into a kjeldahl flask and 15 ml of 40% NaOH solution added to the content in the flask and placed into the decomposition chamber of the distillation apparatus. The condenser tip of the distillation apparatus was then dipped into the boric acid in the conical flask. The ammonia in the solution was then distilled into the boric acid until it changed completely to bluish-green; this lasted at least 15 minutes. The distillate was then titrated with 0.1 N HCl solutions until it became green. A blank was also run through all steps as above. Percent crude protein content of the sample was calculated by using the following formula:

(3) $$\begin{array}{c} \%Crude\;protein=6.25\times \%Nitrogen.\\ \%\text{Nitrogen}=\frac{\left(S-B\right)\times N\times 0.014\times D}{\text{weight of the sample}\times \text{V}}\times 100 \end{array}$$

Where: S = Sample titration reading; B = Blank titration reading; N = Normality of HCl; D = Dilution of sample after digestion; V = Volume taken for distillation; 0.014 = Milli equivalent weight of Nitrogen.

### 3.4. Crude Fat Content

Crude fat was determined using the Soxhlet extraction method. An amount of 2 g of sample was weighed into a muslin thimble. This was inserted into the extraction column with the condenser connected. To extract the crude fat, 200 ml of the extraction solvent (petroleum ether, boiling point 40 - 60) was poured into a previously weighed clean and dry 250 ml round bottom flask and fitted into the extraction unit. The flask was then heated with the aid of electrothermal heater at 60°C for 2 hours. Losses of solvent due to heating were checked with the aid of the condenser so that it cooled and refluxed the evaporated solvent. After extraction, the thimble was removed and the solvent salvaged by distillation. The flask containing the fat and residual solvent was placed on a water bath to evaporate the solvent followed by a further drying in an oven (Wagtech) at 105°C for 30 minutes to completely evaporate the solvent. It was then cooled in a desiccator and weighed. The fat obtained was expressed as a percentage of the initial weight of the sample using the formula:

(4) $$\begin{array}{c} \%\text{Crude fat}=\times 100\%\\ \%\text{Crude fat}=\frac{Weight\;\text{of }fat}{weight\;\text{of }sample}\times 100\% \end{array}$$

### 3.5. Crude Fibre Content

The defatted sample, from crude fat determination, was transferred into a 750 ml Erlenmeyer flask and 0.5 g of asbestos added. A total of 200 ml of boiling 1.25% H_2_SO_4_ was added and the flask immediately set on a hot plate and condenser connected to it. The content was brought to boiling and the sample digested for 30 minutes. At the end of the 30 minutes, the flask was removed and the content filtered through a linen cloth in a funnel and subsequently washed with boiling water until the washing were no longer acidic. The sample was washed back into the flask with 200 ml boiling 1.25% NaOH solution. The condenser again was connected to the flask and the content of the flask boiled for 30 minutes. It was then filtered through the linen cloth and thoroughly washed with boiling water until the washings were no longer alkaline. The residue was transferred to a clean crucible with a spatula and the remaining particles washed off with 15 ml ethanol into the crucible. The crucible with its content was then dried in an oven (Wagtech) at 105°C overnight and cooled in desiccator and weighed. The crucible with its content was then ignited in a furnace (Witeg) at 600°C for 30 minutes, cooled and renewed. The loss in weight gave the crude fibre content and was expressed as a percentage of the initial weight of the sample using the formula.

(5) $$\begin{array}{c} \%\text{Crude fibre}=\times 100\%\\ \%\text{Crude fibre}=\frac{\left(\;\left(wt.\;\text{of }crucible+sample\;before\;ignition\right)-\left(wt.\;\text{of }crucible+ash\right)\right)}{weight\;\text{of }fresh\;sample}\times 100\% \end{array}$$

### 3.6. Carbohydrate Content

Total percentage carbohydrate was determined by adding the total values of crude protein, crude fat, crude fibre, moisture and ash value of the sample and subtracting it from 100. The value obtained is the percentage carbohydrate constituent of the sample.

(6) $$\begin{array}{c} \%\text{Carbohydrate Content}=\left(100-\%\text{moisture}+\%\text{crude protein}+\%\text{crude fat}+\%\text{crude fibre}+\%\text{Ash}\right) \end{array}$$

### 3.7. Phytochemical Screening

Test for Saponins.

To test for saponin, 2 g of the powdered sample was boiled in 10 ml of distilled water for 3-5 minutes. The solution was filtered hot and shaken vigorously. Separation of froth (foam) which persists for some time is an indication of saponins.

#### 3.7.1. Test for Tannins

To conduct tannin test, 0.5 g of the sample was dissolved in 10 ml of distilled water, boiled in a test tube and filtered. Two drops of 0.1% ferric chloride were added and observed for coloration of a blue-black, green or blue-green precipitate which will serve as indication for the presence of Tannins.

#### 3.7.2. Test for Terpenoids

Using the Salkowski test, 0.5 g of the powdered sample extract was added to 2 ml chloroform. After that, 3 ml of concentrated H_2_SO_4_ was added carefully for a reddish-brown colour to be observed.

#### 3.7.3. Test for General Glycosides

About 0.5 g of the powdered sample was placed into two separate beakers and dried at 60°C. After that, 5.0 ml of dilute H_2_SO_4_ was added to one beaker and 0.5 ml of distilled water to the other. The beakers were heated on a boiling water bath for 3 - 5 minutes and the content filtered into two separate test tubes. The filtrates were cooled and neutralized with 3 ml of 5% NaOH solution and the mixture heated with Fehling's solution for 3 minutes. The formation of reddish-brown precipitate in the test tube containing the filtrate from H_2_SO_4_ treatment and the absence of precipitate in the other test tube indicates the presence of glycosides.

#### 3.7.4. Test for Flavonoids

An amount of 0.5 g of the extract was dissolved in 5 ml of 95% ethanol to obtain an alcoholic solution of the sample. Four pieces of magnesium ribbon were added to 3 ml of the ethanoic extract of the sample, after which 3 to 4 drops of conc. HCl was added in a dropwise manner. A Brick-red coloration indicated the presents of flavonoids.

#### 3.7.5. Test for Terpenoids and Steroids

An amount of 0.5 g of the powdered sample was extracted with 5 ml of 95% ethanol; about 1 ml of the ethanoic extract was exposed to dryness in a crucible. It was re-dissolved in 2 ml of chloroform. Two drops of acetic anhydride were added followed by 2 ml of conc. H_2_SO_4_. Reddish-pink colouration indicates terpenoids and steroids.

### 3.8. Statistical Analysis

Sample means and standard errors of the concentrations micro- and macro-minerals as well as readings from the proximate analysis were calculated and expressed as mean ± SE. The raw data obtained were subjected to one-way analysis of variance (ANOVA, Minitab) and t-test where appropriate. Differences were considered to be statistically significant when p < 0.05.

## 4. Results

Analysis Micronutrients in *Solanum torvum.*

The results showed that the *S. torvum* leaves and fruits (either fresh or boiled) contained Cu, Zn, Fe and Mn. There were varied concentrations of these minerals in the leaves and fruits (either boiled or fresh) but these differences were not significant.

The concentration of Cu in the fresh leaves and fruits of *S. torvum* were higher than the concentration in the boiled leaves and fruits (Table 1). Moreover, the concentration of Cu in fresh leaves was higher than the concentration in fresh fruits but these were not significantly different (p = 0.70). Conversely, the concentration of Cu in boiled fruits were higher than that in boiled leaves. However, the difference in concentration was also not statistically significant (p = 0.21). Fresh leaves had numerically higher concentration of Cu than boiled leaves but this was also not significantly different (p = 0.07). Additionally, there was higher concentration of Cu in the fresh fruits than the boiled fruits but the difference was also not significant (p = 0.45). Overall, there were no significant differences in the concentration of Cu between the fresh and boiled leaves and fruits of *S. torvum* (p = 0.123; Table 1).

An assessment of Zn in the fresh leaves and fresh fruits showed that the fresh fruits contained slightly more Zn than the fresh leaves but the difference was not significant (p = 0.80; Table 1). Also, boiled fruits contained slightly more Zn than the boiled leaves but this difference was not significant (p = 0.75). There were no significant differnces in the concentration of Zn in the fresh leaves and boiled leaves (p = 0.80) and in the fresh fruit and the boiled fruits (p = 0.83). The Zn concentrations among the treatments were not significantly different (p = 0.986; Table 1). The results showed varied concentrations of Fe among the boiled and fresh leaves of *S. torvum* but these differences were not significant (p = 0.570; Table 1). There was higher concentration of Fe in the leaves as compared to the fruits. The concentration of Fe was higher in fresh leaves than the fresh fruits and boiled leaves than boiled fruits but the differences observed were both not significantly different (p = 0.73; p = 0.29; Table 1). Also, there were no significant differences in the concentration of Fe in the fresh leaves and boiled leaves of the crop (p = 0.58) and also in the fresh fruits and boiled fruits of the plant (p = 0.51). The concentrations of Mn in the fresh and boiled leaves and fruits of *S. torvum* were not significantly different (P = 0.935; Table 1). The results also showed that Mn concentration in the fresh leaves was not significantly different from the concentration in the fresh fruit (p = 0.72) neither was the concentration of Mn in the boiled leaves significantly different from the concentration in the boiled fruits (p = 0.66). It was also observed that the Mn concentration in the fresh leaves was not significantly different from the boiled leaves (p = 0.54), nor the Mn concentration in the fresh fruit significantly different from the boiled fruit (p = 0.56).

### 4.1. Macro-Minerals in *Solanum Torvum*

The results of the study showed that *S*. *torvum* leaves and fruits contained Na, Mg, P, Ca, K and SO_4_ in the fresh and boiled leaves and fruits of the plants with different concentrations. The concentrations of these macro-minerals though varied in the leaves and fruits either fresh of boiled, were not significantly different. Results from the macro-mineral study on the fresh and boiled leaves and fruits of *S. torvum* showed no significant differences in the concentration of Na (p = 0.630), Mg (p = 0.691), P (p = 0.098), Ca (p = 0.355), K (p = 0.042) and S (p = 0.383), (Table 2). The fresh leaves had lower concentration of Na than the fresh fruit but this variation was not significantly difference (p = 0.24). There was also no significant difference in the concentration of Na in the boiled leaves and the boiled fruits (p = 0.57). The fresh leaves and boiled leaves had almost the same concentration of Na (p = 0.48) and this was not much different between the fresh fruits and the boiled fruit (p = 0.96). The fresh leaves had slightly higher concentration of Mg than the fresh fruit but the difference was not significant (p = 0.94). Meanwhile, the boiled fruits had a higher concentration of Mg than the boiled leaves but this difference was not significant (p = 0.58). The results also showed that the fresh leaves had a higher concentration of Mg than the boiled leaves but not statistically significant (p = 0.31). A similar result was obtained in the case of the fresh fruits and the boiled fruits (p = 0.64). The fresh leaves and fresh fruits had almost the same concentration of P in the plant (p = 0.97). However, the concentration of P in boiled fruits was significantly higher than the concentration in boiled leaves (p = 0.04). In addition, although the concentration of P was higher in fresh leaves than boiled leaves, the difference in concentration was not significantly different (p = 0.13). There was also no significant difference in the concentration of the fresh fruits and the boiled fruits (p = 0.49). The leaves of *S. torvum* had higher concentration of Ca than the fruits. The results from the Ca analysis showed higher concentration of Ca in fresh leaves and fresh fruits but this was not significantly different (p = 0.22). Similarly, the concentration of Ca in boiled leaves was higher than that in boiled fruits but this was not significant (p = 0.26). Results also showed that Ca in the fresh leaves and boiled leaves were almost the same (p = 0.88) and the fresh fruits and boiled fruits also had almost the same concentration of Ca (p = 0.60). The concentration of K in the fresh leaves and fruits of *S. torvum* were almost the same (p = 0.88) but there was a significant difference between the K concentration in the fresh leaves and the boiled leaves of the plant (p = 0.01). Meanwhile, there was no significant difference between the concentration of K in the boiled leaves and boiled fruit (p = 0.11). There was also no significant difference between the concentration of fresh fruits and boiled fruits (p = 0.29). There were no significant differences in the S concentration in the fresh leaves and fresh fruits (p = 0.21) as well as in the boiled leaves and fruits (p = 0.38). Also, there were no significant differences in the concentration of S between the fresh leaves and the boiled leaves (p = 0.62) and between the fresh fruits and the boiled fruits (p = 0.84).

### 4.2. Proximate Analysis

Analysis of the moisture content between the fresh and boiled leaves and fruits of *S. torvum* showed significant differences (p = 0.001; Table 3). The moisture in fresh and boiled leaves was significantly higher than the moisture in fresh fruits. However, the moisture in fresh and boiled leaves was not significantly higher than the moisture in boiled fruits. On the other hand, the fresh fruit and boiled fruits did not show any significant difference in the moisture content (Table 3).

There were no significant differences in the concentrations of carbohydrate (p = 0.227), crude fat (p = 0.183) and ash (p = 0.577) in the fresh and boiled fruits and leaves of *S. torvum* (Table 3).

There were significant differences in the concentration of protein in the fresh and boiled fruits and leaves of *S. torvum* (p < 0.001). The protein in the fresh and boiled leaves was significantly higher than that in the fresh and boiled fruits (Table 3).

There were variable concentrations of crude fiber in the samples of *S. torvum* (p = 0.023; Table 3).

Phytochemical analysis showed that the fresh fruits, boiled fruits, fresh leaves and boiled leaves contained saponins, tannins and glycosides but the extraction of glycosides with H_2_SO_4_ was negative for the boiled fruit (Table 4). Flavonoids and Terpenoids/Steroids were present in both the fresh and boiled leaves but absent in the fresh and boiled fruits.

## 5. Discussion

This study has shown that the fruits and leaves of *S. torvum* are rich in diverse nutrients and phytochemicals, some of which have been reported to be good for the improvement of blood conditions and boosting of the immune system [6, 9, 19, 20, 27]. The formation of red blood cells and other properties of the blood is dependent on the concentration of Fe and other minerals in the blood. It is reported that the recommended dietary allowance (RDA) of Fe is 8 mg/day in boys and girls and up to 18 mg/K in adult with a tolerable upper intake level of 45 mg/day [28, 29]. Our findings indicate that *S. torvum* contains high concentrations of Fe in both fruits and leaves. In our findings, we had more than twice higher and three and half times higher in Fe concentration in the boiled fruits and leaves of *S. torvum,* respectively, than what has been reported [15]. This supports the traditional belief that the consumption of *S*. *torvum* help in the management of anaemia especially in pregnant women, nursing mothers and children thus the use in food to improve health. The findings also support an earlier study by Serfor-Armah et al. [30] which reported that *S. torvum* plants had higher concentration of Fe than the fruits. This implies that, the leaves of *S. torvum* will be more efficient in the management of Fe deficiency in pregnant women and children than the fruit. The current study also showed that the leaves of *S. torvum*, either fresh or boiled contained more Fe than the fruits, making it a better source of Fe than the fruit. The high Fe content in the plant is good for the improvement of hemoglobin in the blood to prevent anemia [15, 20]. This is probably the reason why traditional health practitioners use fruits and leaves of this plant to manage anemia and other ailments [15, 19]. With this knowledge, it will be better for nutritionist, mothers and traditional health practitioner to use leaves of *S. torvum* more often than fruits to manage anaemic conditions. Based on our findings, under critical anaemic conditions, ingestion of *S. torvum* leave juice may give a quicker solution than juice from fruit because of the Fe concentration. The study also showed that boiling does not have much effect on the concentration of Fe in *S. torvum*. Copper, as a trace mineral is a component of the antioxidant enzyme glutathione peroxidase ([31–34]. Copper is essential but in high doses it affect biological processes, cause anaemia, liver and kidney damage, stomach and intestinal irritation [34, 35]. The estimated average requirement (EAR) per day ranges from 260 *μ*g/day in children to 900 *μ*g/day in men and women with an upper limit of 10 mg/day [28]. Comparing the concentration of Cu in a Kg of the *S. torvum* to the estimated daily requirement and the quantity of the plant used in the preparation of food and juice, there is very minimal risk of getting high dose of Cu from the consumption of *S. torvum*. There are reports that Cu is used for treatment of stomach ulcers because of how it combines with anti-inflammatory and anti-bacterial properties [36] The Cu content of fresh fruits and leaves of turkey berry were higher in this study than Cu concentration of cabbage in earlier studies [37]. The boiled leaves had a lower copper concentration than cabbage. This implies that the consumption of *S*. *torvum* can help miminize Cu deficiency and improve mother and child nutrition in developing countries. Comparing the quantity of *S. torvum* added to a meal it can be said that there is minimal risk of take over dose of copper if one eats *S. torvum*. It is safe to eat *S. torvum* than cabbage because of pesticides and other agrochemicals used by farmer of cabbage which may be mutagenic or cancerous. Furthermore, *S. torvum* can be found freely in the wild or bought at a cheaper cost than cabbage so the poor can afford. This suggests that *S. torvum* can serve as a secondary source of Cu in diet. Zinc plays a key role in maintaining vision [38], and it is present in high concentrations in the eye [39]. Low Zn levels can be associated with male infertility and sickle cell disease [40]. There was Zn in *S. torvum* which can help to supplement the daily need from food. The concentration in the leaves and fruits either fresh or boiled were almost the same. There was no significant difference between the concentrations of *S. torvum* among the treatment means. Zinc is required in maintaining the proper functioning of the immune system, protein synthesis, wound healing, DNA synthesis and cell division [41]. This implies that the consumption of *S*. *torvum* by the people help to reduce the risk of contraction certain diseases and enhance growth of the individuals especially children and pregnant women. For instance, the amount of Zn detected in turkey berry in this study was higher than the amount detected in cabbage [37]. Therefore, nutritionists should encourage people to add *S. torvum* to their diet to improve nutrient gain, enhance good health and improve vision. People should also eat turkey berry and obtain the same concentration of Zn as green pepper. Manganese is important in connective tissues, formation of bones and blood-clotting factors. It is also involved in fat and carbohydrate metabolism, Ca absorption and blood sugar regulation [42, 43]. In this study, the concentration of manganese in fresh leaves and fruit, and boiled leaves and fruits of *S. torvum* were relatively the same. The highest concentration of manganese was recorded in fresh fruits. The presence of manganese in the plant helps to prevent some of the permanent organ damage if consumed by the pregnant woman or children [2]. Macro-minerals in the plant included Ca, Mg, S, P, Na and K. Potassium, Ca and Mg are said to serve as electrolytes in the blood plasma [19]. The electrolytes help to retain water and prevent dehydration of organisms. This makes the *S. torvum* a good source of nutrient to enhance blood conditions in organisms. The percentage concentration of P in the fresh leaves and fruits of *S. torvum* were not different. However, the concentration of P was significantly different between boiled fruits and leaves. The highest mean concentration of P was detected in boiled fruits and the lowest mean concentration was in boiled leaves of *S. torvum*. It is therefore proposed that, people with hypophosphatemia could be given boiled fruits of *S. torvum* to enable them raise the amount of phosphate in their blood. Calcium is important for the development, growth and maintenance of bones [44]. It was observed in this study that, leaves of *S. torvum* had more Ca than fruits. Therefore, Ca-deficient persons could consume leaves of *S. torvum* to augment the amount of Ca in their body. In fact, Ca is a major dietary element essential for strong bones and teeth formation. Magnesium is necessary to maintain the health of muscles, including the heart, and for the transmission of electrical signals in the body. Magnesium aids in muscle relaxation during activity and also assists in producing certain proteins and energy [45]. It is necessary for the release of parathyroid hormone and for its action in the backbone, kidney and intestine and for the reactions involve in converting vitamin D to its active form. Fresh leaves and fruits of *S. torvum* had the highest percentage concentration of Mg which was the same in both. This implies that one will get the same benefit from using either the fresh leave or fruits of *S. torvum*. However, the percentage concentration of Mg was higher in the fresh leaves and fruits of *S. torvum* than the boiled leaves and fruits. This confirms the traditional belief that the use of the fresh leaves and fruits gives quicker response than the boiled on. It is therefore advisable to use the fresh leaves and fruit to achieve better result. There was a decrease in the magnesium content when heat was applied to turkey berry. On the other hand, there was no significant difference in magnesium when it was boiled. Potassium is recommended for maintaining proper fluid balance and cardiac (heart muscle) function as well as maintaining the balance of acids and bases in the body. Moreover, K is also important for muscle contractions [46]. The percentage concentration of K in *S. torvum* in this study was high in fresh leaves and fruits of *S. torvum* than in the boiled leaves and fruits. These differences can be attributed to boiling which might have resulted in nutrient lost to the stock. It is therefore advisable to use the stock to maximize nutrient grain by people. Sodium is one of the common and widely used inorganic elements. It is responsible for performing various key functions such as nerves and muscle function and ensuring a right balance of fluids in the body. The boiled fruits of *S. torvum* recorded the highest percentage concentration of Na while the fresh leave recorded the least percentage concentration. The difference observed in the both fruits and leaves either fresh or boiled were not significant. This implies that people may eat the fruit or leaves either fresh or boiled and get the same effect. Sulphate (SO_4_^2-^) is an essential macro element which form part of amino acids, connective tissues, skin, hair and nails. [47, 48]. The content of sulphate was highest in fresh leaves of *S. torvum* followed by boiled leaves with fresh fruit recording the least concentration. The percentage concentration of SO_4_ was higher in the leaves (fresh and boiled) than that of the fruit (fresh and boiled). Moisture content of foods gives indication of dry matter available in the food and its major role in food analysis is to determine the shelf life of the food [49]. The study recorded mean moisture which agrees with the findings of Akoto et al. [15] who reported moisture of 86% in *S. torvum* fruit. The high moisture content of *S. torvum* in the varied forms (raw and boiled) of the fruits and leaves of *S. torvum* shows that the samples have limited shelf life. The moisture content of boiled fruits was slightly higher than that of the raw fruits and the moisture content in boiled leaves was higher than the fresh leaves. This implies that *S. torvum* cannot be stored for a long time in the forms used for the study though the fresh fruits and leaves was significantly different and was less than the moisture in the boiled fruits and leaves. The variation seen between the raw and boiled samples were a result of boiling. It can therefore be deduced that the fresh fruit and leaves have a longer shelf life than the boiled fruits and leaves. It is therefore advisable to store the fruits and leaves in the fresh form than boiled. High moisture content in the samples studies supports the results of previous studies [15, 50]. The results showed mean crude fat higher than the finding of earlier studies [15, 50]. Protein content in the leaves (fresh and boiled) was higher than that of the fruits (fresh and boiled). The findings of our study do not vary much from the mean protein recorded by Ogah [50] but was found to be at least four times higher than the results obtained by Akoto et al. [15] in *S. torvum* fruit. The findings from the study support those of Ogah [50] which recorded the highest protein in raw turkey berry than in the cooked ones. Indicating that consumption of *S. torvum* leaves especially when fresh will be a great source of protein essential for human growth and function than the fruit either fresh or boiled. Percentage of the Ash in a sample gives an idea on the inorganic content of the sample from where the mineral content could be obtained. Ash content observed in our study was lower than what Ogah [50] and Akoto *et al*. [15] reported. There was no significant difference in the ash content in the various samples though fresh leaves was high. Plants derive their colour and some level of protection from phytochemicals. In terms of protection, phytochemicals prevent plants from invasion of animals, diseases and infection. Nevertheless, animals also derive some benefits from phytochemicals. Phytochemicals build the immune system of animals and control the growth of cells to prevent cancer. Our study shows that the phytochemicals, saponin, tannins, glycosides, flavanoids and steroids are present in *S. torvum.* These phytochemicals are known to boost the immune system and are helpful for the proper metabolic functioning of the body [51]. Our findings are in line with a previous study [9] which showed that plants in the Solanum genus contain phytochemical including saponin, polyphenols and glycosides in the leaves and berries. The presence of the phytochemicals in the *S. torvum* we studied suggests that they could be a good source of antioxidant to improve nutrition. Our findings also showed that boiling did not have effect on the phytochemicals in the leaves. It is worth noting that, both fresh and boiled fruits of *S. torvum* did not contain flavonoids and terpenoids/steroids. Meanwhile, the leaves of some *Solanum* species contain flavonoids and glycosides as their major phytochemicals [9]. This could be why consumers of other *Solanum* species in sub-Sahara Africa prefer the leaves of the plant to the fruit [24]. Contrary to this, the people in Ghana use the fruits of *S. torvum* in meals and use the leaves only for medicinal purposes (JD. Abraham, per. obs.). It is believed that the leaf is more potent for the management of anaemic conditions than the fruit. The fresh leaves are usually preferred by medicinal users than the fruit.

## 6. Conclusions

*Solanum torvum* has different micro and macro-minerals, protein, fat, carbohydrate and phytochemicals which are needed for the metabolic functioning of the body. The micro-nutrients in *S. torvum* include Fe, Zn, Cu and Mn and the macro-minerals include P, SO_4_, Ca, Mg, Na and K. It also contains phytochemicals such as saponins, flavonoids and glycosides. The use of the fruits and leaves in food preparation could help to increase the overall nutritional content of the consumed food and improve health. Therefore, the consumption of *S. torvum* is encouraged by this paper. Turkey berry is recommended for incorporation in the dishes cooked for pregnant women, nursing mothers and children in schools and hospitals to boost their nutrient intake since it is highly nutritious. The nutrients and phytochemicals contained in *S. torvum* are helpful for the proper metabolic functioning of the body. However, it was observed that boiling has effect on the concentrations of some of the nutrients and phytochemicals in the plant although the effect was mostly not significant. The saponins in the plant would help to reduce the risk of cancer and the accumulation of blood lipids in the individual. This would help improve the immune system and prevent future disease conditions.

## Figures and Tables

**Table 1:** Mean ± SE Concentration (mg/Kg) of micronutrient in *Solanum torvum* (n = 6).

Constituent	Fresh fruit	Boiled fruit	Fresh leaves	Boiled leaves
Cu	14.97 ± 1.84	12.90 ± 1.85	16.27 ± 2.65	8.63 ± 2.60
Zn	16.58 ± 1.40	16.15 ± 1.36	16.024 ± 1.60	15.11 ± 2.88
Fe	208.43 ± 18.51	192.57 ± 13.96	226.50 ± 46.82	274.23 ± 67.37
Mn	102.83 ± 17.26	91.23 ± 7.02	95.2 ± 11.20	99.13 ± 15.55

Means did not show any statistical difference in micro-element concentration at P < 0.05.

**Table 2:** Mean ± SE Concentration (%) of macro-mineral in *Solanum torvum.*

Constituent	Fresh fruit	Boiled fruit	Fresh leaves	Boiled leaves
Na	0.31 ± 0.05	0.32 ± 0.08	0.24 ± 0.03	0.27 ± 0.03
Mg	0.32 ± 0.06	0.29 ± 0.03	0.33 ± 0.05	0.25 ± 0.05
P	0.58 ± 0.08	0.66 ± 0.07	0.58 ± 0.10	0.34 ± 0.11
Ca	0.47 ± 0.07	0.42 ± 0.06	0.69 ± 0.15	0.74 ± 0.24
K	1.84 ± 0.21	1.55 ± 0.14	1.80 ± 0.13	1.26 ± 0.09
SO_4_	1.5 ± 0.15	1.44 ± 0.24	2.11 ± 0.41	1.84 ± 0.35

No significant differences were observed in the concentrations of the various macro-minerals in all the treatments.

**Table 3:** Mean ± SE Nutritional composition (%) of *Solanum torvum.*

Constituent	Fresh fruit	Boiled fruit	Fresh leaves	Boiled leaves
Moisture	79.81 ± 0.49^**b**^	83.06 ± 1.34^**a,b**^	84.43 ± 1.11^**a**^	86.03 ± 0.40^**a**^
Carbohydrate	0.33 ± 0.03	0.34 ± 0.02	0.29 ± 0.03	0.37 ± 0.02
Crude fat	8.81 ± 1.46	7.86 ± 1.18	5.25 ± 0.98	6.82 ± 0.61
Protein	10.72 ± 0.51^**b**^	12.50 ± 0.40^**b**^	17.05 ± 1.08^**a**^	16.05 ± 0.58^**a**^
Ash	0.06 ± 0.004	0.06 ± 0.004	0.06 ± 0.01	0.07 ± 0.01
Crude fibre	25.12 ± 1.92^**a**^	24.16 ± 0.56^**a,b**^	16.27 ± 2.21^**b**^	20.08 ± 2.34^**a,b**^

Means with different letters attached in rows indicate statistical difference at P < 0.05.

**Table 4:** Phytochemical screening of *Solanum torvum* fruit and leaves.

	Saponins	Tannins	Flavonoids	Terpenoids/steroids	Lycosides	
					H_2_SO_4_	H_2_O
Fresh fruits	+	+	_	_	+	+
Boiled fruits	+	+	_	_	_	+
Fresh leaves	+	+	+	+	+	+
Boiled leaves	+	+	+	+	+	+

Positive (+) means present and negative (-) means absent of constituent.

## Data Availability

The data used to support the findings of this study are included within the supplementary information file.

## Additional Points

*Practical Application*. The findings of this research will help nutritionists to improve the diet of anaemic patients, pregnant women and nursing mothers. It will also help improve the prevention and management of micronutrient deficiency.

## Supplementary Materials

Supplementary Materials Data supporting Table 1. Concentration (mg/Kg) of micronutrient in *Solanum torvum*. Data supporting Table 2. Concentration (%) of macronutrient in *Solanum torvum*. Data support Table 3. Nutritional composition (%) of Solanum torvum. Data supporting Table 4. Phytochemical screening of *Solanum torvum* fruit and leaves.
