# Supplementary material for: Effect of Boiling on the Nutrient Composition of Solanum Torvum
Source: Int J Food Sci. 2022 Mar 16;2022:7539151. doi: 10.1155/2022/7539151 (PMC8942660; doi:10.1155/2022/7539151)
Supplement: Supplementary Materials — Data supporting Table 1. Concentration (mg/Kg) of micronutrient in Solanum torvum. Data supporting Table 2. Concentration (%) of macronutrient in Solanum torvum. Data support Table 3. Nutritional composition (%) of Solanum torvum. Data supporting Table 4. Phytochemical screening of Solanum torvum fruit and leaves. [file 7539151.f1.pdf]

Effect of boiling on the nutrient composition of *Solanum torvum*

Janice Dwomoh Abraham, Emmanuel Sekyere, Isaac Gyamerah

Supplementary data

Data supporting Table 1. Concentration (mg/Kg) of micronutrient in *Solanum torvum*

Cu (mg/Kg)

Replicate	Fresh leaves	Fresh fruits	Boiled leaves	Boiled fruits
1	10.2	14.6	17	8.8
2	26.4	15.6	2.6	11
3	17.6	17	8.8	21.2
4	10.2	19.4	6.6	10.2
5	12.6	16.8	15.2	14.8
6	20.6	6.4	1.6	11.4

Zn (mg/Kg)

Replicate	Fresh leaves	Fresh fruits	Boiled leaves	Boiled fruits
1	10.26	16.4	25.62	15.28
2	19.64	12.7	9.48	9.94
3	16.02	18.14	14.1	17.16
4	16.1	20.56	11.42	16.86
5	13.32	19.36	21.8	19.3
6	20.8	12.34	8.26	18.36

Fe (mg/Kg)

Replicate	Fresh leaves	Fresh fruits	Boiled leaves	Boiled fruits
1	117	173.2	554	184.4
2	295.6	197.4	142	216.4
3	274.2	235	250.6	210.6
4	130.4	263.4	162	126.6
5	141.4	238.6	385	211.6
6	400.4	143	151.8	205.8

Mn (mg/Kg)

Replicate	Fresh leaves	Fresh fruits	Boiled leaves	Boiled fruits
1	63.2	91.8	136.2	101.6
2	127.4	90	62.2	74.4
3	102	111.8	92.6	117
4	103.6	178.2	92.6	71.6
5	60.4	95.6	152.2	87
6	114.6	49.6	59	95.8

Data supporting Table 2. Concentration (%) of macronutrient in *Solanum torvum*

% Na

Rep	Fresh leaves	Fresh fruits	Boiled leaves	Boiled fruits
1	0.1746	0.2716	0.2056	0.1142
2	0.3718	0.151	0.2202	0.2226
3	0.2344	0.2708	0.293	0.378
4	0.1924	0.3206	0.426	0.6012
5	0.205	0.3246	0.2362	0.4274
6	0.2436	0.547	0.2356	0.1722

% Mg

Rep	Fresh leaves	Fresh fruits	Boiled leaves	Boiled fruits
1	0.228	0.399	0.266	0.399
2	0.494	0.228	0.19	0.19
3	0.418	0.38	0.19	0.266
4	0.342	0.532	0.19	0.304
5	0.152	0.304	0.494	0.209
6	0.342	0.095	0.19	0.361

% P

Rep	Fresh leaves	Fresh fruits	Boiled leaves	Boiled fruits
1	0.4841848	0.6326056	0.7330048	0.569712
2	0.5273312	0.4474184	0.1423488	0.7681872
3	0.2668336	0.925848	0.1069904	0.5244184
4	0.5229576	0.4257088	0.4472336	0.4650184
5	0.984544	0.6405168	0.4972352	0.899888
6	0.669416	0.4150344	0.1005576	0.7112864

% Ca

Rep	Fresh leaves	Fresh fruits	Boiled leaves	Boiled fruits
1	0.384	0.384	1.344	0.384
2	0.832	0.384	0.256	0.192
3	0.96	0.448	0.512	0.448
4	0.32	0.768	0.448	0.384
5	0.448	0.512	1.6	0.64
6	1.216	0.32	0.256	0.48

% K

Rep	Fresh leaves	Fresh fruits	Boiled leaves	Boiled fruits
1	1.7398	1.6632	1.435	1.5336
2	1.7184	1.4626	1.4876	1.2958
3	1.4814	2.7986	1.3064	1.1866
4	1.615	1.623	0.9196	1.6096
5	1.7994	1.9516	1.3336	2.1616
6	2.423	1.5163	1.051	1.532

% SO_4

Rep	Fresh leaves	Fresh fruits	Boiled leaves	Boiled fruits
1	1.36	1.04	1.48	1
2	2.6	2.12	1.44	1.04
3	1.4	1.72	2.96	0.76
4	1.12	1.36	2.88	2.28
5	3.68	1.36	1.32	1.84
6	2.52	1.4	0.96	1.72

Data support Table 3. Nutritional composition (%) of *Solanum torvum*

Sample	Replicate	Moisture	Ash	protein
Fresh fruit	1	79.88	0.067033	10.5
	2	78.2	0.062734	12.5125
	3	78.63	0.046498	10.7625
	4	81.34	0.05424	11.6375
	5	80.76	0.060355	9.8875
	6	80.02	0.069939	9.0125

Sample	Replicate	Moisture	Ash	protein
Boiled fruit	1	78.14	0.071582	12.25
	2	80.89	0.060195	11.025
	3	83.66	0.057853	12.775
	4	83.6	0.048118	13.3875
	5	87.86	0.0695	11.9
	6	84.19	0.050592	13.65

Sample	Replicate	Moisture	Ash	protein
Fresh leaves	1	84.4	0.050188	21.875
	2	86.53	0.085466	15.1375
	3	83.69	0.091705	17.5
	4	79.57	0.030941	16.625
	5	87.18	0.049444	14.2625
	6	85.2	0.056496	16.8875

Sample	Replicate	Moisture	Ash	protein
Boiled leaves	1	84.73	0.075363	17.625
	2	86.74	0.068869	15.4
	3	85.88	0.079554	17.5
	4	86.85	0.079554	14.875
	5	86.95	0.073853	14.2625
	6	85.02	0.043714	16.625

Sample	crude fat	crude fibre	carbohydrate
Fresh fruit	12.135	24.515	0.26298
	5.05	20.12	0.30128
	8.56	26.68	0.34403
	9.485	29.155	0.38973

Sample	crude fat	crude fibre	carbohydrate
Boiled fruit	7.455	25.06	0.30065
	11.3	22.89	0.32125
	6.095	25.155	0.33475
	6.57	23.54	0.3968

Sample	crude fat	crude fibre	carbohydrate
Fresh leaves	4.62	12.505	0.2842
	7.46	13.795	0.31473
	2.88	22.44	0.3568
	6.04	16.32	0.21585

Sample	crude fat	crude fibre	carbohydrate
Boiled leaves	7.54	14.66	0.32095
	5.82	25.595	0.40445
	5.755	21.74	0.38835
	8.18	18.32	0.36185

Data supporting Table 4. Phytochemical screening of *Solanum torvum* fruit and leaves

	Phytochemistry				Glycosides	
**Sample ID**	**Saponins**	**Tannins**	**Flavonoids**	**Terpenoids / Steroids**	**H2SO4**	**H2O**
Fresh fruits 1	positive	positive	negative	negative	Positive	positive
Fresh fruits 2	positive	positive	negative	negative	Positive	positive
Fresh fruits 3	positive	positive	negative	negative	Positive	positive
Boiled fruits 1	positive	positive	negative	negative	negative	positive
Boiled fruits 2	positive	positive	negative	negative	negative	positive
Boiled fruits 3	positive	positive	negative	negative	negative	positive
Fresh leaves 1	positive	positive	positive	positive	Positive	positive
Fresh leaves 2	positive	positive	positive	positive	Positive	positive
Fresh leaves 3	positive	positive	positive	positive	Positive	positive
Boiled leaves 1	positive	positive	positive	positive	Positive	positive
Boiled leaves 2	positive	positive	positive	positive	Positive	positive
Boiled leaves 3	positive	positive	positive	positive	Positive	positive
